# The early intestinal immune response in experimental neonatal ovine cryptosporidiosis is characterized by an increased frequency of perforin expressing NCR1^+^ NK cells and by NCR1^−^ CD8^+^ cell recruitment

**DOI:** 10.1186/s13567-014-0136-1

**Published:** 2015-03-11

**Authors:** Line Olsen, Caroline Piercey Åkesson, Anne K Storset, Sonia Lacroix-Lamandé, Preben Boysen, Coralie Metton, Timothy Connelley, Arild Espenes, Fabrice Laurent, Françoise Drouet

**Affiliations:** Department of Basic Sciences and Aquatic Medicine, Faculty of Veterinary Medicine and Biosciences, Norwegian University of Life Sciences, Oslo, Norway; Department of Food Safety & Infection Biology, Faculty of Veterinary Medicine and Biosciences, Norwegian University of Life Sciences, Oslo, Norway; Institut National de la Recherche Agronomique, UMR1282, Infectiologie et Santé Publique, Laboratoire Apicomplexes et Immunité Muqueuse, Nouzilly, France; The Roslin Institute, Royal (Dick) School of Veterinary Studies, University of Edinburgh, Edinburgh, UK

## Abstract

*Cryptosporidium parvum*, a zoonotic protozoan parasite, causes important losses in neonatal ruminants. Innate immunity plays a key role in controlling the acute phase of this infection. The participation of NCR1+ Natural Killer (NK) cells in the early intestinal innate immune response to the parasite was investigated in neonatal lambs inoculated at birth. The observed increase in the lymphocyte infiltration was further studied by immunohistology and flow cytometry with focus on distribution, density, cellular phenotype related to cytotoxic function and activation status. The frequency of NCR1+ cells did not change with infection, while their absolute number slightly increased in the jejunum and the CD8+/NCR1- T cell density increased markedly. The frequency of perforin+ cells increased significantly with infection in the NCR1+ population (in both NCR1+/CD16+ and NCR1+/CD16- populations) but not in the NCR1-/CD8+ population. The proportion of NCR1+ cells co-expressing CD16+ also increased. The fraction of cells expressing IL2 receptor (CD25), higher in the NCR1+/CD8+ population than among the CD8+/NCR1- cells in jejunal Peyer’s patches, remained unchanged during infection. However, contrary to CD8+/NCR1- lymphocytes, the intensity of CD25 expressed by NCR1+ lymphocytes increased in infected lambs. Altogether, the data demonstrating that NK cells are highly activated and possess a high cytotoxic potential very early during infection, concomitant with an up-regulation of the interferon gamma gene in the gut segments, support the hypothesis that they are involved in the innate immune response against *C. parvum*. The early significant recruitment of CD8+/NCR1- T cells in the small intestine suggests that they could rapidly drive the establishment of the acquired immune response.

**Electronic supplementary material:**

The online version of this article (doi:10.1186/s13567-014-0136-1) contains supplementary material, which is available to authorized users.

## Introduction

As with all neonatal mammals, the new-born ruminant is challenged by infections at vulnerable mucosal sites like the gut mucosa, frequently leading to enteritis. *Cryptosporidium parvum* (*C. parvum*), a protozoan parasite highly prevalent in cattle and small ruminant flocks throughout the world is a zoonotic agent. In sheep, *C. parvum* causes moderate to severe, but usually self-limiting enteric neonatal disease [1,2] with low mortality. However, in very young ruminants, this parasite may cause profuse diarrhoea and can lead to death by dehydration if combined with co-infections or deficiencies in nutrition and husbandry [3]. The parasite cycle ends with either thin-walled oocysts that auto-infect the host or thick-walled oocysts that are released in the environment [4]. Both animal health and welfare, economic impact and the zoonotic aspect make cryptosporidiosis one of the most important gastro-intestinal diseases in ruminant production. To date there is no vaccine available, and halofuginone lactate is the only drug with marketing authorization for preventive treatment of cryptosporidiosis [5,6]. To develop an adequate immunoprophylaxis strategy, it is therefore important to clarify the early immune events leading to a protective response against this parasite as neonates frequently become infected within the few hours following birth.

Only limited information is available on the neonatal ruminant intestinal immune response to *C. parvum* during the early stages of the infection. Pathogenicity and brief pathology of ovine cryptosporidiosis were described in lambs for the first time [1,2,7] more than three decades ago and more recent data were obtained in calves describing the intestinal response to the parasite with an increase of T cell subsets [8-12]. Nevertheless, our understanding of the immuno-pathological response to *C. parvum* remains poor in these species.

Recovery and protection from reinfection have been associated with a CD4+ T cell response starting from the second week post inoculation [13-15]. In cattle, this response has been associated with a production of gamma interferon (IFNγ) [11,12]. SCID mice lacking B and T cells develop chronic inflammation upon *C. parvum* infection, which progressively becomes fatal [13,15,16]. More recent experiments performed with mice tend to demonstrate that the innate immune system could be sufficient to resolve the infection [17] and we recently showed in neonatal mice that innate immunity can control the acute phase of the disease [18]. As Natural Killer (NK) cells are key players in innate immune responses they might play a role in the early host immune response against this parasite in young lambs. NK cells have been suggested to be important participants in the immune response against *C. parvum* infection; Barakat et al. [19] found that NK cells had an important role for the innate control of *C. parvum* infection in mice and Dann et al. [20] showed that NK cells lead to clearance of cryptosporidia from the intestine of humans.

Most of the studies on the role of NK cells in *C. parvum* infections have been performed with adult murine models which are not the most suitable species for studying *C. parvum* pathogenesis; indeed they are not naturally susceptible, rarely develop diarrhoea and do not develop the same mucosal pathology as observed in larger animals and humans [21,22].

The jejunum and ileum contain Peyer’s patches (PPs) that are considered as immune sensors of the intestine and are important for immune protection at mucosal surfaces and the induction of mucosal immune responses in the intestine [23,24]. Whereas the PPs of the jejunum (JPPs) are recognized as secondary lymphoid organs of the intestinal wall, the continuous ileal PP (IPP) is also responsible for the generation of B cells and is thus considered as a primary lymphoid tissue [25-28]. The specialized follicle associated epithelium (FAE) that overlies PPs is capable of transporting luminal antigens [29] to the underlying immune cells to promote a tolerogenic or an inflammatory response, which will be set in action in the lamina propria. Our aim was to get an insight into the early local immune response in the different sections of the small intestine and associated lymphoid tissues of lambs during the neonatal period with a particular focus on NK cells, which we have shown to be active in neonatal calves [30], and CD8 T lymphocytes, that have been shown to be important in controlling *C. parvum* infection in humans [31].

In lambs inoculated soon after birth, we observed an activation of the NCR1+ NK population in the gut with increased expression of perforin, CD16 and CD25. In contrast, the expression of perforin and CD25 by CD8+/NCR1- T lymphocytes did not increase in infected lambs although the density and percentages of this population increased from day 3 post-inoculation (pi) in both the inductive and effector sites of the small intestine.

## Materials and methods

### Animals and experimental design

The lambs used for this study were born from Préalpes ewes maintained in protected facilities with a conventional status (PFIE-INRA-37380 Nouzilly). At birth the lambs were allowed to suckle the colostrum and then received artificial milk *ad libitum* until euthanasia. Within 24 h, age-matched “pairs of lambs” (occasionally triplets), i.e. lambs born within a 12 h interval, were relocated to two identical rooms, one for the inoculated lambs and one for the controls. The day following birth, the animals were inoculated *per os* with 2 × 10^6^ oocysts of *C. parvum* (day 0 pi). During the experiment, symptoms were registered and pathological signs briefly recorded at the time of slaughter. Animals were slaughtered at various days pi (dpi), i.e. 0, 1, 2, 3, 6 and 11 dpi by electric stunning and bleeding according to the AMVA guideline on euthanasia; matched pairs of lambs were slaughtered the same day and their organs processed simultaneously. All experimental protocols were conducted in compliance with French legislation (Décret: 2001–464 29/05/01) and EEC regulations (86/609/CEE) governing the care and use of laboratory animals, after validation by the local ethics committee for animal experimentation (CEEA VdL: 2011-05-2).

### Parasite and infection

#### Collection of oocysts

*C. parvum* oocysts were isolated from the faeces of neonatal calves infected with oocysts initially obtained from an infected child and maintained by repeated passage in calves. Oocysts were purified as previously described [32].

#### Parasitic load detection

**Figure 1:**
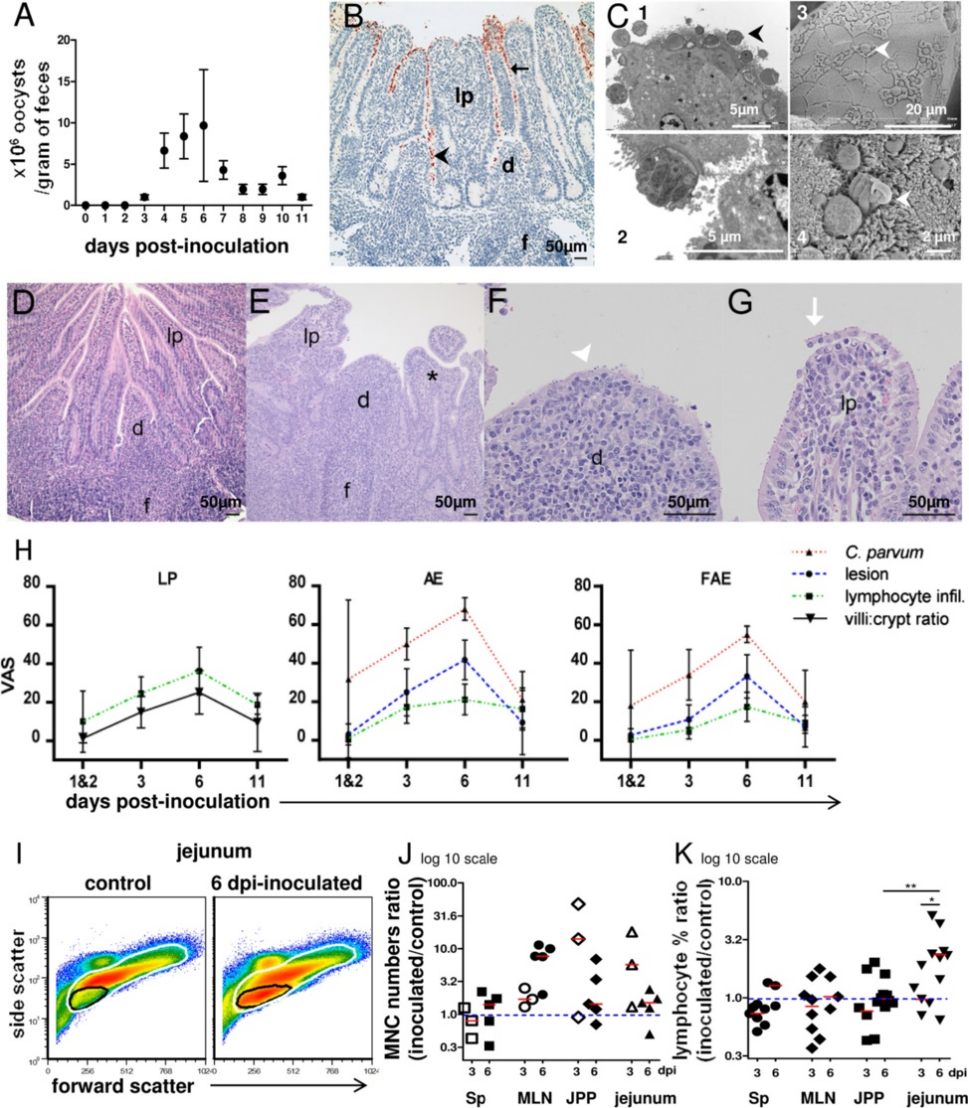
***C. parvum*** **oocyst excretion, lesions and lymphocyte infiltration during infection. (A)** *C. parvum* oocyst excretion: mean/gram of faeces ± standard error (10 to 15 animals). **(B)** *C. parvum* parasitic stages immunolabelled in brown in a 3 dpi ileal Peyer’s patch (IPP): on the brush border of absorptive epithelium (AE) (arrow) and follicle-associated epithelium (fae/FAE) (arrowhead) covering the lamina propria (lp/LP) and dome (d). Lymphoid follicle (f). **(C)** Trophozoites and meronts at 6 dpi, in the JPP AE (arrowhead) observed by transmission EM (C1, C2) and scanning EM of meronts in infected FAE (C3; arrowhead) and merozoite leaving a meront (C4; arrowhead). **(D-G)** PPs representative changes during infection (HE staining). JPPs of control **(D)** and 6 dpi inoculated lambs **(E)** with villous atrophy and fusion (*) and lymphocyte infiltration in lp. **(F-G)** Attenuation, lymphocyte infiltration, detachment of fae (arrowhead) and absorptive epithelium (AE) (arrow) in an infected IPP. **(H)** Semi quantitative scoring of histopathological changes in IPP during infection. Sections from control lambs were used as a baseline and each change was rated according to severity on a 0 to 100 visual analogue scale (VAS). Values represent mean with 95% confidence intervals for 4 to 8 animals. **(I-K)** Cells extracted of spleen (Sp), mesenteric lymph nodes (MLN), JPPs and jejunum from matched pairs of lambs were purified on density gradients and mononuclear cell (MNC) and lymphocyte percentages were determined by flow cytometry on morphology parameters. **(I)** Plots show the gating of lymphocytes (black gate) and MNC (white gate) in the jejunum. In this example, lymphocytes represented 11 and 38% of MNC (88 and 84% of the cells analyzed) in control versus matched inoculated lamb, respectively. **(J)** The MNC absolute number ratio and **(K)** the lymphocyte percentage ratio were determined for each pair of lambs at 3 or 6 dpi. The red bars indicate the median values. Mann–Whitney statistic test: significance * *p* < 0.05, ** for *p* < 0.01.

In a set of animals, faeces were collected daily to assess the oocyst excretion pattern (Figure 1A). The first oocysts pass in the faeces at day 3 or 4 pi. Therefore, to assess the parasitized status of the inoculated lambs slaughtered early after inoculation (before parasite excretion) and the uninfected status of their controls, the presence of *C. parvum* in the mucosa was tested on fragments of intestine by assessing the expression of a cryptosporidium-specific gene by real time RT-PCR as previously described [32]. From day 4 pi the level of infection was also assessed by counting oocysts in the faeces as described by Naciri et al. [5].

### Collection of tissue specimens

Samples of jejunum, JPPs, IPP, spleen and small intestinal mesenteric lymph nodes (MLN) were taken. Tissue sampling for cryostat sectioning was performed as previously described [33]. In short, tissues were chilled in isopentane before freezing in liquid nitrogen and storage at −70 °C. In addition, tissues were fixed in formalin and embedded in paraffin wax (FFPE). Some tissues were snap frozen in liquid nitrogen for RNA analyses. In pairs of age matched control and inoculated lambs, fresh tissues were collected in ice-cold RPMI medium supplemented with 5% foetal calf serum (FCS) and 1% penicillin streptomycin (P/S) for extraction of the cells.

### Antibodies used for labelling

The antibodies (Abs) used in this study were against: ovine NCR1/NKp46 (EC1.1; IgG1 [34]), bovine NCR1/NKp46 (AKS6; IgG2b [35]), bovine TcR1-N7 (86D; IgG1) that labels γδ-T lymphocytes, bovine CD25 (CACT116A; IgG1), from VMRD/WSU (Pullman, USA), human CD16 (KD1; IgG2a, [36,37]), human CD3 (A0452; pAb) from Dako (Trappes, France), ovine CD8 (38.65; IgG2a) from Serotec (UK) and Ki67 (ab15580; pAb and NCL-L-Ki67-MM1; IgG1) from Abcam (Cambridge, UK) and Novocastra Laboratories- Leica (UK) respectively. The anti-human perforin-FITC kit (δG9; IgG2b) was from BD Pharmingen (France). IgG1, IgG2a, IgM mouse isotype controls for flow cytometry were from Dako and IgG2b from Caltag- Invitrogen (France). Isotype controls for IHC against IgG1, IgG2a and IgG2b were from BD Biosciences (USA). Subtype-specific secondary Abs conjugated with Tricolor (TC) or R-Phycoerythrin (PE) were from Caltag. Goat anti-mouse IgG Fab’2 secondary Abs conjugated with Fluo Probe (FP) 488 were from Fluo Probes- Interchim (France). Alexa Fluor-conjugated secondary Abs AF 350, 488, 546, 594 and 633 for indirect immunofluorescence were from Molecular Probes- Invitrogen.

### Histology techniques

All FFPE tissues were stained and examined with haematoxylin and eosin (H&E) according to standard histological techniques [38] for routine histological examination. Samples of intestine for electron microscopy (EM) were fixed with 3% glutaraldehyde in 0.1 M cacodylate buffer, then processed as previously described for transmission EM [39] and scanning EM [40]. The samples were examined with a Jeol 1010 transmission electron microscope (Jeol, Croissy-sur-Seine, France) and a FEG Gemini 982 scanning electron microscope (Carl Zeiss, Jena, Germany). For in situ immunolabelling, standard indirect methods with avidin-biotin complex peroxidase (Vectastain® ABC Kit, Vector Laboratories, USA) were used against *C. parvum*. Prior to immunolabelling of the FFPE sections, 4 μm thick sections were placed on positively charged slides and dried at 59 °C. After a standard dewaxing procedure, sections were treated for antigen retrieval in citrate buffer (0.01 M citric acid monohydrate, pH 6.0) in a microwave. Endogenous peroxidase was inhibited by treatment with 3% H_2_O_2_ in methanol for 10 min. Further blocking of unspecific binding and incubation with antibodies were performed as described by the manufacturer. The specific binding of the antibodies was visualized by using ImmPACT™ AEC after counter staining with Mayer’s haematoxylin. Indirect immunolabelling was performed on cryosections, according to a protocol described earlier [33,41]. Fluorescent sections were examined under a Leica DM RXA fluorescence microscope (Germany), and images were captured using a SPOT RT Slider^TM^ camera (Diagnostic Instruments, USA) with SPOT 5.0 Advanced Software (Diagnostic Instruments). In addition, images were captured using a Zeiss Axiovert 100 inverted microscope, equipped with an LSM 510 laser confocal unit with the Zeiss ZEN 2009 Software (Carl Zeiss).

### Microscopic evaluation of immuno-labelled slides

To ascertain whether morphological features observed on H&E sections were related to *C. parvum* infection in the gut, the sections were blind coded. Features present in each section were listed and each feature was subjectively recorded in a visual analogue scale (VAS) ranging from 0 to 100. For immunofluorescent qualitative analysis of NCR1+ cells, single-blinded analysis was done. For quantitative analysis of CD8+ cells in the IPP, images were taken and processed as previously described [33]. Briefly, images of 400× from at least 5 individual villi and domes from the ileal segment were taken. A pixel-to-millimetre calibration was performed and the areas were defined in mm^2^.

### Extraction and purification of mononuclear cells from the organs

Spleen and MLN tissues were processed as previously described [42]. The whole organs were treated to assess the absolute number of mononuclear cells (MNC). All mediums and chemicals were from Sigma-Aldrich (Lyon, France) unless otherwise stated. Briefly, the tissues were disrupted in Hanks medium (HBSS) containing 2% FCS and 1% P/S by crushing on a 200 μm nylon gauze with a syringe piston. Splenic red blood cells were lysed with ammonium chloride solution (0.155 M NH4Cl, pH 7.4) then resuspended in HBSS medium. MNC were then purified on Histopaque^TM^ d = 1.077, washed and stored in ice cold RPMI medium supplemented with 10% FCS and 1% P/S until labelling.

Gut tissues were processed with a technique adapted from Renaux et al. [43] and Pérez-Cano et al. [44] to recover lamina propria MNC. Briefly, the gut tract was emptied of faecal content and rinsed with Phosphate Buffer Saline (PBS) buffer. The JPPs were dissected carefully, pooled then processed as the jejunal tissue. The whole jejunum and the JPPs were weighed separately before 30-gram samples of jejunum were taken for extraction of cells. The gut was opened and cut into 1 cm^2^ fragments. The epithelial cells and intra-epithelial lymphocytes were extracted by incubation for 20 min at 37 °C in HBSS without Ca and Mg containing 3 mM ethylene diamine tetraacetic acid disodium salt (EDTA), 2 mM dithioerythritol, 10% FCS and 1% P/S under magnetic stirring and discarded. Then the lamina propria lymphocytes were extracted. The EDTA treated intestinal pieces were washed with HBSS, then incubated at 37 °C for 45 mn under magnetic stirring in RPMI medium containing 9.25U/mL type I collagenase, 30U/mL dispase II (Roche, Rosny sous Bois, France) and 2500U/mL bovine pancreas DNase I (Calbiochem, USA). The cell suspension was filtered on a 500 μM nylon mesh and the cells were washed with RPMI-10% SVF. The MNC were purified on a 75%/40% Percoll (GE Healthcare - Bio-Sciences, Sweden) gradient, washed and stored in ice cold RPMI-10% FCS until labelling. The living cells were counted with Thoma chambers and the absolute number in the organ (spleen, MLNs or JPPs) was calculated as follows: number of cells per mL multiplied by number of mL of cell suspension for the whole organ. For the jejunum, as the cells were extracted from 30 grams of tissue, the latter result was multiplied by the ratio: jejunum total weight (in grams)/30. For all the analyses and comparisons reported, the organs from age-matched control and inoculated pairs of lambs were processed simultaneously to minimize technical induced variations.

### Cell labelling and flow cytometry

Single or multiple indirect labelling of surface receptors was performed on purified ovine cells using Abs against the molecules NCR1, CD8, TCR1, CD16 and CD25 revealed by subtype-specific secondary Abs. Direct intracellular perforin labelling was performed with the perforin-FITC kit and the Cytofix/Cytoperm and Permwash solutions (BD Pharmingen). The samples were analysed on a FACS CALIBUR flow cytometer (Becton Dickinson), equipped with Cell-Quest Pro software. At least 2 × 10^5^ viable cells were analysed for spleen, MLN and PPs and at least 10^6^ cells for the jejunum. The analyses of labelling were performed with the FCS express software; percentages of perforin+ and CD25+ cells and median fluorescence intensity (MFI) were determined using the histogram subtraction and statistics functions of the software. Relative numbers of cell subsets determined by flow cytometry (subset %) were converted into absolute numbers per organ according to the formula: (subset% × MNC%)/100 × total number of cells in organ (as calculated above).

### RNA isolation and real-time RT-PCR

RNA was extracted from tissues with TRIZOL solution (Invitrogen), according to the manufacturer’s instructions. Purified RNA was reverse-transcribed using oligo (dT) primers and M-MLV reverse transcriptase (Promega, France). For PCR experiments, primer pairs were designed using Primer 3 software (Additional file 1). Each primer was designed on different exons to span the intervening intron and thus avoid amplification from contaminating genomic DNA. Q-RT-PCR assays were carried out by combining cDNA with primers and IQ SYBRGreen Supermix (Bio-Rad, USA) and were run on a Chromo4 (Bio-Rad). Samples were normalized internally using the average cycle quantification (Cq) of three reference genes Hypoxanthine phosphoribosyltransferase (HPRT), b-Actin and Glyceraldehyde-3-phosphate dehydrogenase (GAPDH). Gene expression values are expressed as relative values after Genex macro analysis (Bio-Rad).

### Statistical analyses

For the morphometric analysis of the density of CD8+ cells, it was necessary to compensate for natural variability between individuals; therefore the non-parametric Wilcoxon-van Elteren [45] test was used to calculate the significance of differences between the two groups. Two-tailed tests were performed and differences considered significant for *p*-values < 0.05. Flow cytometry data were analysed with GraphPad software: the nonparametric Mann–Whitney test was used to test the significance of differences between means from inoculated lambs and matched controls, the Wilcoxon test to compare different subsets of cells from the same animals and the paired *t* test to compare groups of paired lambs.

## Results

### *C. parvum* induces typical enteritis lesions with immune cell recruitment in the segments of the neonatal ovine small intestine

In neonatal lambs infected on the day of birth, the kinetics of *C. parvum* oocyst excretion was evaluated daily (Figure 1A). The *C. parvum* infected or uninfected status was verified by measuring the expression of a cryptosporidium specific gene in gut tissues by RT-PCR (data not shown). The excretion of oocysts started from day 3 pi coinciding with the onset of watery diarrhoea and mild dehydration and reached a peak at 6 dpi (Figure 1A). At necropsy, there was a mild to moderate enlargement of mesenteric lymph nodes (MLN) in the inoculated lambs from day 2 pi (1.5-3 fold weight increase on average compared to controls) while no visible change of the spleen was observed. Histopathological observations confirmed the macroscopical findings demonstrating a mild to moderate, diffuse and catarrhal enteritis. Parasites were observed in the brush border throughout the whole gut, with increased density in distal jejunum and ileum. In all PPs, the FAE was also infected (Figures 1B, 1C-F) but, as previously observed in the mouse model (unpublished data), we could not observe developing parasites in M cells. The lesions of superficial, lymphocytic and granulocytic diffuse enteritis, with shortening and bridging of the villi (Figures 1D-G) affected not only the absorptive epithelium, but also the FAE of the dome in which attenuation and sloughing and, to a lesser degree necrosis, could be observed. The pathological changes and the amount of *C. parvum* found on the epithelium increased gradually, reaching a peak at 6 dpi, and were partly resolved by day 11 pi (Figure 1H). In gut sections from JPPs and IPP, the mononuclear cell (MNC) infiltration (identified mainly as lymphocytes with small round and dense nuclei surrounded by narrow eosinophilic cytoplasm) was observed from day 3 pi in both villi and dome and their respective epitheliums (Figures 1E-H). Using flow cytometry, we analysed the recruitment of lymphoid cells in MLNs, the jejunum and JPPs, and spleen as a reference of a systemic organ (Figures 1I-J). There was a tendency towards an increase in the absolute number of MNCs in the gut and MLNs at 3–6 dpi: the ratio between the absolute numbers of MNCs in inoculated lambs and their matched controls was superior to one in most pairs i.e. 6 of 8 in JPPs, 7 of 8 in the jejunum and 8 of 8 in the MLNs (Figure 1J). This was consistent with the necropsy and histology findings, and suggested that the immune response to this infection occurs locally and within the draining lymph nodes. Considering the percentage of total lymphocytes in the different tissues, we found that in most (10 of 13) pairs of lambs at 3 and 6 dpi, there was an increase in the lymphocyte proportion in the jejunum of the inoculated lamb (ratio significantly superior to 1, *p* < 0.01) (Figure 1K) and this local increase could already be observed as early as 1 and 2 dpi (data not included in Figures 1J and K).

**Figure 2:**
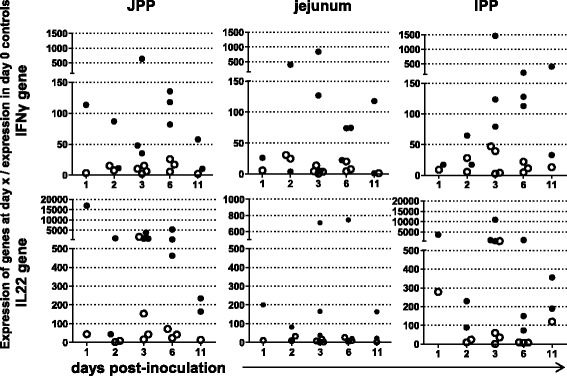
**IFNγ and IL22 gene expression in samples from intestinal tissues.** At slaughter, fragments of the small intestine were frozen in nitrogen and processed for quantification of IFNγ and IL22 gene expression by qRT-PCR. Each point represents the ratio of the number of gene copies in one individual (Ct) to the reference corresponding to the mean of the number of copies in two control animals slaughtered at birth (Ct0). The open and filled circles correspond to control and inoculated lambs respectively. JPP; jejunal Peyer´s patch, IPP; ileal Peyer´s patch.

We and others have shown that interferon gamma (IFNγ) is a key cytokine for controlling infected enterocytes in the mouse model [32,46]. In contrast, interleukin 22 (IL22), that is now considered to play an important role in intestinal tissue repair [47,48], has not been investigated in response to *C. parvum* infection. Infection of lambs was associated with an increase in the expression of the IFNγ and IL22 genes which was evident from as early as 1 dpi with a further increase observed at 3 and 6 dpi (Figure 2). The up-regulation of IFNγ and IL22 during the infection may participate in the clearance of the infection and the recovery of the epithelial integrity, respectively.

### The proportion of NCR1+ lymphocytes did not change with infection although a slight increase in their absolute numbers was observed in the small intestine of infected lambs

NCR1+ lymphocytes are known to be IFNγ producing cells and important players of the innate response. As an increase of lymphocytes in the gut segments was observed early in the infection at 3 and 6 dpi (Figures 1E-H, K) and even earlier at 1–2 dpi (data not included in Figure 1K), we analysed the local changes of NCR1+ lymphocytes in the GALTs at different time-points of infection. Preliminary studies revealed that the proportion of NCR1+ cells was rather low in the ileum compared to jejunum and JPPs; consequently immunohistology was preferred to flow cytometry to assess the cell recruitment in the IPP.

**Figure 3:**
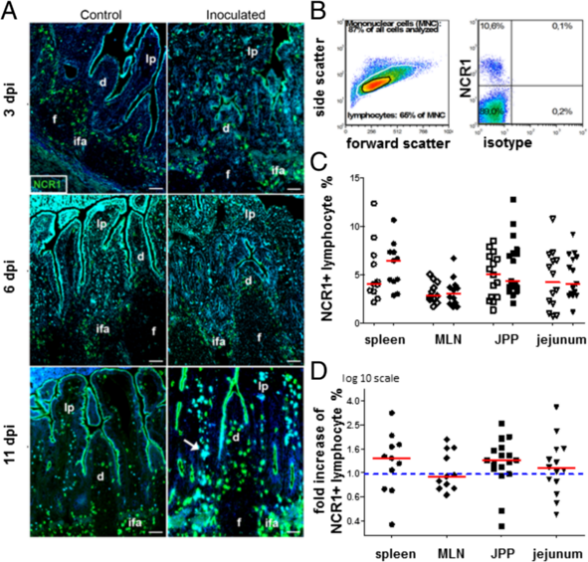
**Proportions of NCR1+ lymphocytes in different tissues during** ***C. parvum*** **infection. (A)** Sections from ileal Peyer’s patches of lambs 3, 6 and 11 dpi and their age-matched controls were labelled with NCR1 (green) mAb. NCR1+ cells changed neither in density nor location during infection. Diffuse light blue spots were identified as autofluorescence using the blue filter, especially in the lamina propria (lp). Dome (d), interfollicular area (ifa), follicle (f). Bar 50 μm. **(B-D)** The NCR1+ lymphocyte percentage within the mononuclear cell population (MNC) was determined by flow cytometry. **(B)** The plots show the example of the NCR1 labelling in cells purified from jejunal Peyers’patches (JPP) at 6 dpi and gated as indicated (black gate); the right plot shows the labelling with both the anti-ovine NCR1 mAb and the isotype control of the CD8 mAb. **(C)** The NCR1+ lymphocyte percentage is shown in the different organs of lambs from 1 to 6 or 7 days of age: each dot represents one animal and the bars indicate the mean values. Control lambs (open symbols) inoculated lambs (filled symbols). Mesenteric lymph nodes (MLN). **(D)** The points represent the fold increase of NCR1+ lymphocytes (i.e. ratio inoculated/control NCR1+ lymphocyte percentage) for each pair of age-matched lambs shown in graph C, and the red bars show the medians. The mean ratio was significantly superior to one with a confidence interval of 1.21-1.57 for spleen, 1.05-1.15 for MLN, 1.17-1.55 for JPP and 1.28-1.34 for jejunum (*p* < 0.01).

As previously found in the GALTs of other healthy 1–2 month-old lambs [33,34,49], NCR1 labelling of IPP sections (Figure 3A) revealed the presence of NCR1+ cells in the interfollicular T cell areas and domes, and to a lesser degree in the lamina propria and intraepithelial compartment, while few or none were observed in the follicles. There was neither a difference in the density (number of labelled cells per area) nor a change in localization of NCR1+ cells in the infected lambs compared to the controls at any time during the infection (Figure 3A). The NCR1+ lymphocyte proportions were also examined by flow cytometry in gut segments, MLN and spleen (Figures 3B-D). No difference in the proportion of NCR1+ lymphocytes (Figure 3C) or in the percentages ratio (inoculated/matched control) (Figure 3D) could be detected in any organ at any time-point. Also, no difference was detected in the level of expression of the NCR1 gene in the different gut segments (Additional file 2). However, both the absolute number of mononuclear cells and the lymphocyte percentage were higher in the jejunum of inoculated lambs (Figures 1H, J, K). In addition, when specifically examining the absolute numbers of NCR1+ lymphocytes in gut segments of paired lambs, a statistics test could only be applied to data from JPPs at day 6 pi (*p* < 0.05), but a similar tendency to an increase in infected lambs was seen at other time points and in the jejunum (Additional file 3). We conclude that in small intestinal segments, NCR1+ lymphocytes do not increase in relative numbers, although they most likely increase in absolute numbers during the early stages of infection.

### The frequency of NCR1+ lymphocytes expressing perforin increases with infection and is concurrent with a preferential increase of the NCR1+/CD16+ subpopulation in JPPs and jejunum

**Figure 4:**
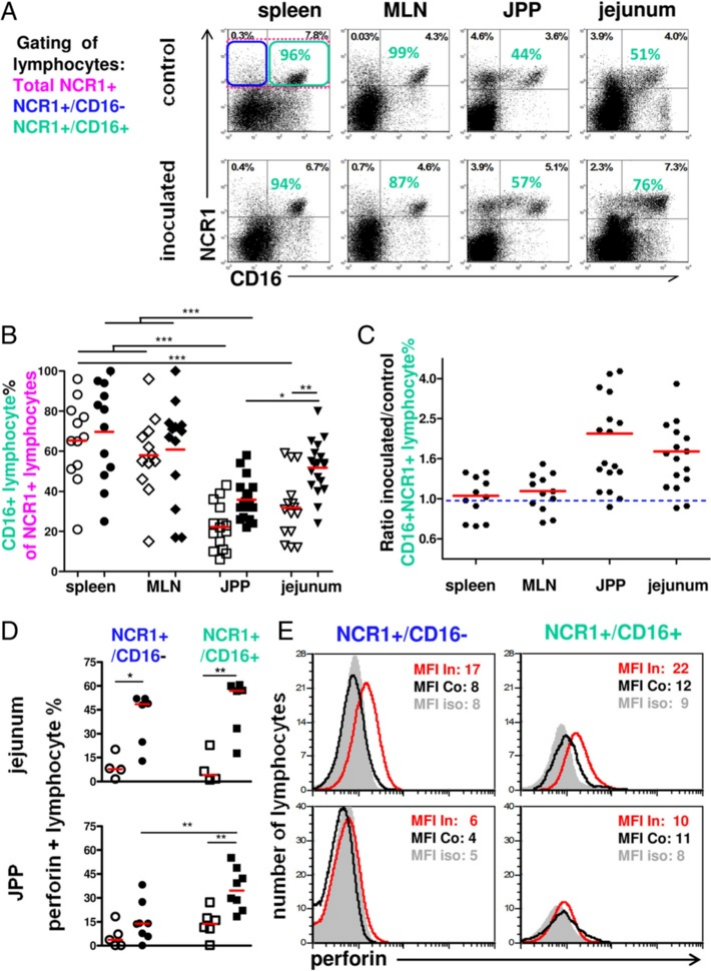
**Increase in NCR1+/CD16+ lymphocyte percentage and perforin+ cell percentage in the jejunum during infection. (A)** Cells labelled with anti NCR1 and CD16 mAbs and gated on the lymphocyte gate (shown in Figure 1I) were analyzed by flow cytometry in spleen, mesenteric lymph nodes (MLN), jejunal Peyer’s patches (JPP) and jejunum. The quadrants were set according to the labelling obtained with the isotype controls for the same number of cells analyzed. Percentages indicated in black represent the CD16-/NCR1+ and CD16+/NCR1+ lymphocyte percentages of which the non-specific labelling was subtracted. Percentages of CD16+ cells among NCR1+ lymphocytes are indicated in green. Data are from an inoculated lamb at 6 dpi and its control. **(B)** Individual CD16+ percentages of NCR1+ lymphocytes are indicated for animals 2–6 dpi, control lambs (open symbols), inoculated lambs (filled symbols). The red bars indicate the mean values. **(C)** The points represent the inoculated/control ratio of NCR1+/CD16+ lymphocytes for each pair of age-matched lambs. **(D-E)** NCR1 and CD16 double labelled cells were permeabilized for intracellular labelling with an anti-human perforin mAb or isotype control to determine the perforin+ cell percentage in both NCR1+ subpopulations by histogram subtraction at 6 dpi. Control lambs (open symbols), inoculated lambs (filled symbols). **(E)** The data shown are representative of the lambs whose individual percentages of perforin+ cells are shown in Figure 4D. The red and black line limited histograms correspond to the inoculated lamb (In) and its matched control (Co) respectively, the gray filled histogram to the isotype control of the perforin mAb (iso); the mean fluorescence intensity (MFI) is indicated for each histogram. The comparisons were made with the Mann–Whitney (control/inoculated) and Wilcoxon tests (paired data). Significant difference probability: * *p* < 0.05, ** *p* < 0.01, *** *p* < 0.001. The inoculated/control CD16+/NCR1+ lymphocyte ratio was calculated for each pair of lambs **(C)**; the mean was significantly superior to 1 with confidence interval of 1.4-2.82 for JPPs and 1.48-2.32 for jejunum (*p* < 0.01).

Of the two subsets of NK cells described in humans, the CD56^dim^CD16+ population is considered to have strong cytotoxic properties. Recently, the presence of two populations of NK cells with different CD16 expression were described in the sheep intestine [33,50]. Since the expression of CD16 might reflect the cytotoxic potential of NK cells, we analysed the balance between the NCR1+/CD16+ and NCR1+/CD16- subpopulations during the course of infection (Figures 4A-C). In control animals more than 55-60% NCR1+ lymphocytes expressed the CD16 receptor in spleen and MLN (Figure 4B) while, in JPPs and jejunum, they represented only 20-40% of NCR1+ lymphocytes (Figure 4B). In inoculated animals an increase in the NCR1+/CD16+ subpopulation (2 fold on average) was observed at 3–6 dpi in the cells extracted from JPPs and jejunum (*p* < 0.01) (Figure 4C). In addition, in around half of the inoculated lambs an increase in the level of expression of the CD16 receptor was observed (MFI), (data not shown).

The perforin content, which directly reflects the cytotoxic potential of the NCR1+ lymphocytes, was examined in the same two subpopulations at 6 dpi (Figure 4D). In both subpopulations, the percentage of perforin+ cells (Figure 4D) and their mean perforin content (MFI shown in the representative example (Figure 4E)) were increased in infected lambs.

### *C. parvum* infection induces a strong increase of the CD8 + NCR1- cell population in the intestine

**Figure 5:**
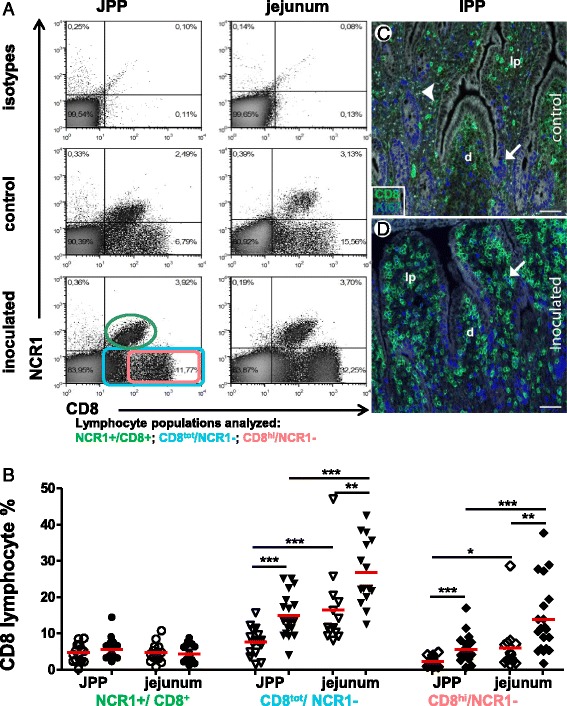
**CD8+ lymphocyte increase in the jejunum and jejunal Peyer’s patches. (A)** Cells isolated from jejunal Peyer’s patches (JPP) and jejunum were double labelled with anti CD8 and NCR1 mAbs and analyzed by flow cytometry. The plots show data from a 6 dpi inoculated lamb and its 7 day-old matched control and are representative of 8 matched pairs of 7 day-old lambs. The gating used for the analyses shown in B is indicated with the corresponding colours. **(B)** The percentages of the 3 different populations of CD8+ lymphocytes corresponding to the 3 gates shown in **(A)** were analyzed in jejunum and JPP from lambs 2–6 dpi. Control lambs (open symbols), inoculated lambs (filled symbols). The means are indicated in red. The comparisons were made with the Mann–Whitney (control/inoculated) and Wilcoxon tests (paired data). Statistically significant differences are indicated with * p < 0.05, ** *p* < 0.01, *** *p* < 0.001. **(C-D)** Section of an ileal Peyer’s patch (IPP) from a 7 day old control **(C)** and a 6 dpi lamb **(D)** were double labelled with anti CD8 (green) and Ki-67 (blue) antibodies. The images shown are obtained by merging these images with the white light image. The double positive cells (arrow) display CD8 cytoplasmic staining and Ki67 nuclear staining. Pinpoint sized spots (arrowhead) are identified as autofluorescence. Dome (d), lamina propria (lp). Bar 50 μm.

**Figure 6:**
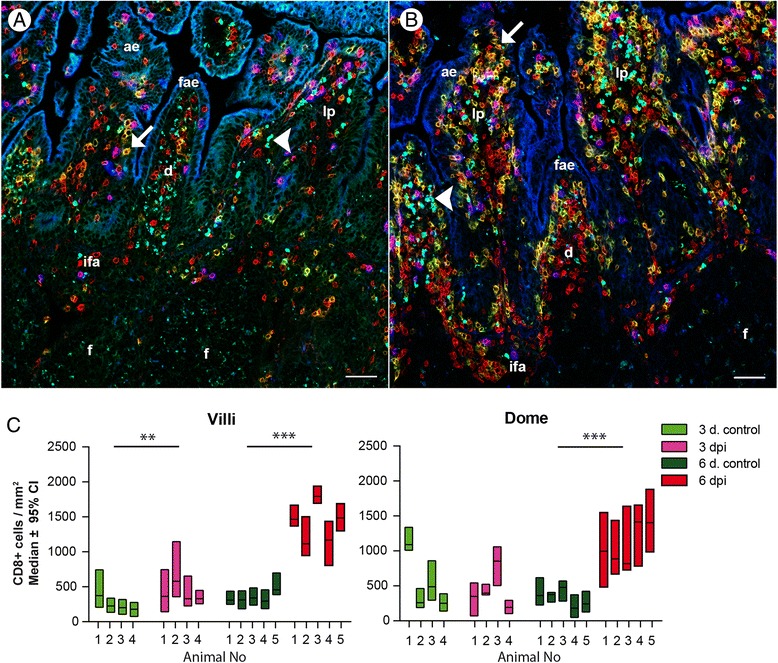
**Significant increase of CD8+ cells in the ileum of infected lambs.** Ileal Peyer’s patches sections from a pair of 6 day-old matched lambs, control **(A)** and inoculated **(B)**, were labelled with Ab against CD3 (red), CD8 (green) and γδTCR (blue). Increased numbers of yellow CD3+/CD8+ cells (arrow) in the lamina propria (lp) and dome (d) were seen in the inoculated animals compared with the controls. Few or no CD3+/CD8+/γδTCR+ cells were observed. Light blue dots (arrowhead) were identified as autofluorescence and thus differentiated from the specific labelling which was localised in the cell membrane. Absorptive epithelium (ae), follicle-associated epithelium (fae), follicle (f), interfollicular area (ifa). Bar 50 μm. **(C)** The increase of CD3+/CD8+ cells in the ileal Peyer’s patch was demonstrated by quantitative analysis of positive cells per mm^2^ in villi and dome, including the covering epithelium. The graphs show the median with 95% confidence interval constructed using the Bernoulli-Wilcoxon procedure. Statistically significant differences are indicated as ** *p* < 0.01 and *** *p* < 0.001.

Circulating NCR1 lymphocytes from ruminants are known to express the CD8 marker, also expressed by subsets of both αβ and γδ T lymphocytes [30,34,51]. We therefore examined the expression of this marker in JPPs and jejunum and found that the vast majority of NCR1 lymphocytes expressed CD8 (Figure 5A). In addition, a large population of CD8+ NCR1- lymphocytes was also observed (Figure 5A). Three populations of CD8+ lymphocytes could be distinguished: NCR1+/CD8+ NK cells, CD8^tot^/NCR1-, with a CD8^hi^/NCR1- cell population included in the latter. The proportion of NCR1+/CD8+ NK cells was similar in JPPs and jejunum and did not change during the infection (Figure 5B), which was in agreement with the results shown in Figures 3C and D. However, the CD8^tot^/NCR1- cells that already predominated in JPPs and particularly in the jejunum at homeostasis increased significantly during infection (3–6 dpi); this increase was not due to a specific increase of the included CD8^hi^/NCR1- sub population (shown in Figure 5A). We sought to elucidate if this global increase of the CD8^tot^/NCR1- population was caused by a recruitment or local proliferation of CD8+ lymphocytes. Double immunofluorescent labelling against CD8 and Ki-67 on gut sections (Figures 5C and D) showed that although the density of CD8+ cells increased in the inoculated lambs, the proportion of CD8+/Ki-67+ proliferating cells remained the same as in the controls, indicating that only a minor fraction of the local CD8+ cell population was proliferating in situ and that the increase in the CD8+ cells most probably was caused by cells recruited from blood. As several cell types may express CD8, we sought to clarify the identity of the CD8+ cells in sections. In gut sections, the vast majority of CD8+ cells were also CD3+ (Figures 6A and B) and thus represent CD8 T lymphocytes. As the CD8^low^ labelling of NCR1+ cells was not perceptible in immunohistology (not shown), we conclude that we only could visualize the CD8^high^ population of the NCR1- cells with this technique. Their density increased in villi, dome and their respective epitheliums in both JPPs and IPP. As the changes were observed to be similar in the jejunal and ileal segments, IPP was chosen for quantification to verify the observations in the sections (Figure 6C). CD8+ cell density increased significantly at days 3 and 6 pi, in the lamina propria and absorptive epithelium of the villi and at 6 dpi in the dome including FAE which supported the flow cytometry data (Figure 5B). To discriminate between αβ and γδ T cells and to better characterize the CD8+/NCR1- population, we analyzed the expression of TCR1, the γδ T cell receptor, on CD8+ cells. TCR1+ T cells were scarce in gut sections of JPPs and IPP (Figures 6A and B), and no obvious increase of density was observed in infected animals compared to controls. Similar results were found with flow cytometry (Additional file 4) although this method indicated a 2–3 fold increase of γδ T cell percentage in jejunum. Only 3 to 12% of CD8+ cells co-expressed TCR1. Thus the vast majority of CD8+/NCR1- lymphocytes recruited during the infection are most probably conventional αβ CD8+ T cells.

### The proportion of perforin+ cells within the intestinal NCR1+ population and their activation (CD25 MFI) increase with infection while they remain stable in the CD8+/NCR1- population

**Figure 7:**
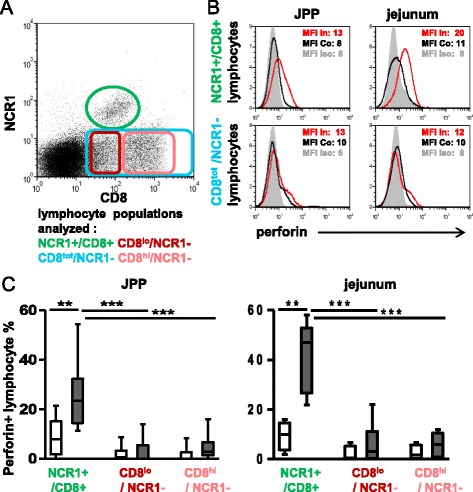
**Perforin+ lymphocytes among CD8+ lymphocytes in the gut** ***.*** **(A)** Cells extracted from gut tissues were double labelled with anti NCR1 and CD8 mAbs then fixed and permeabilized and labelled with an anti perforin mAb. The plot shows (example of jejunum at 6 dpi) the gating of the lymphocyte populations analyzed. **(B)** Lymphocytes from an inoculated lamb (3 dpi) and its control were gated either on the CD8+/NCR1+ or the CD8^tot^/NCR1- populations then analyzed for the perforin content. The red line limited histogram corresponds to the inoculated lamb (In), the black line limited histogram to its matched control (Co) and the gray filled histogram to the isotype control of the perforin mAb (iso) and the mean fluorescence intensity (MFI) is indicated for each histogram. **(C)** The analysis of the perforin expression by CD8+ lymphocyte sub-populations (gates shown on Figure 7A) was performed in 3 control and 4 age-matched 6 dpi inoculated lambs. The comparisons were made with the Mann–Whitney (control/inoculated) and Wilcoxon tests (paired data). Statistically significant differences are indicated with ** *p* < 0.01 and *** *p* < 0.001.

**Figure 8:**
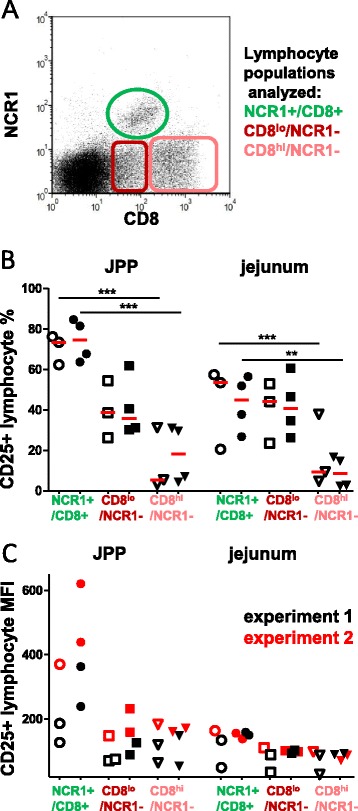
**Expression of CD25 on small intestinal NCR1+ and CD8+ lymphocytes. (A)** The expression of the activation marker CD25 was analyzed at 6 dpi in the populations gated as indicated. The individual percentages **(B)** and the mean fluorescence intensity (MFI) of CD25+ cells **(C)** are shown for the 3 populations. Medians are shown with red bars. Control lambs (open symbols), inoculated lambs (filled symbols). In C, the results of the two experiments are shown with red or black symbols. The comparisons were made with the Mann–Whitney (control/inoculated) and Wilcoxon tests (paired data). Statistically significant differences are indicated with ** *p* < 0.01 and *** *p* < 0.001.

To determine the functional potential of the CD8+ subpopulations, the perforin content (Figure 7) and the activation status (Figure 8) were analyzed by flow cytometry. The percentage of perforin+ cells among the NCR1+/CD8+ population was already noticeably increased in both JPPs and jejunum while it was low in the CD8+/NCR1- population in the age-matched pair tested at 3 dpi (Figures 7A and B). At 6 dpi, this feature was confirmed (Figure 7C). The activation status of the same populations was also assessed through the expression of the IL2 receptor (CD25) (Figure 8). In JPPs of control animals, around 70% the NCR1+ lymphocytes expressed CD25 compared with 40% of the CD8^lo^ and less than 30% of the CD8^hi^ population. This latter population was also the less activated in the jejunum. Surprisingly, the proportion of CD25+ cells did not increase during the infection in any of the populations analysed. However, considering the higher level of expression of the CD25 marker (MFI) on the NCR1+ lymphocytes of JPPs from the inoculated lambs at day 6 pi, these cells were significantly more activated than in the controls (Additional file 5).

## Discussion

The early immune response of neonatal ruminants to *C. parvum* infection is still largely unknown although some insight has been gained on the recruitment of CD8 lymphocytes by Wyatt et al. [10,52] and of mast cells by Li et al. [53] in the gut of infected calves. Natural Killer (NK) cells have been known for a long time to be important actors of the primary innate immune response through cytotoxicity and IFNγ production and also through a regulatory role in the immune response via their interaction with other cells and their ability to produce various cytokines once activated. The objective of this work was to investigate the participation of NK cells in the first steps of the innate immune response to *C. parvum* in a natural host, the neonatal lamb, in response to a controlled experimental infection. NK cells express several receptors, including CD8, that are common to several lymphocyte types. Over the last decade, the activating receptor NKp46, renamed Natural Cytotoxicity Receptor 1 (NCR1) in the new nomenclature, had come to be considered as the prototypal marker to define NK cells in most species [35,54,55]. However, various recent studies on innate immune cells in mice and humans led to the discovery that NCR1+ cells not only include conventional NK cells (cNK) but also innate lymphoid cells (ILC) of groups 1 and 3 (including NK22/ILC22) [56]. In mouse and human, ILCs participate in the intestinal defence and homeostasis but, to date, these cells have not been characterized in ruminants and their presence in this species is therefore still speculative. We examined the changes in the NCR1+ lymphocytes, in two inductive sites of the small intestine, the ileal (IPP) and jejunal Peyer’s patches (JPPs), both known for playing distinct roles in the mucosal immunity of sheep, and the jejunum which is considered a major effector site with a special focus on days 3 and 6 pi [28,57,58]. The two techniques used for this study (immunohistology and flow cytometry) brought concordant and complementary results. The histopathological findings revealed a marked lymphocyte infiltration and proliferation in the intestinal mucosa already by day 3 pi (end of the parasite prepatent period) and at day 6 pi (around the peak of infection). Some Ki-67+ proliferating cells were CD8+ cells suggesting a proliferation in situ beside recruitment from blood and secondary lymphoid organs. CD8 is expressed both by T cells and most mucosal NK cells in ruminants, and both of these lymphocytes are known to be recruited in inflammatory conditions and several infections in human and mice [59,60]. NCR1+/CD8+ lymphocytes representing NK cells were part of the early lymphocyte infiltration observed in MLN and all the small intestine and the CD3+/CD8+/NCR1- population (corresponding to CD8+ T lymphocytes) also presented a marked increase in the lamina propria and absorptive epithelium of the villi and, with a short delay, within the dome and FAE of inoculated lambs during this period as previously observed by Wyatt et al. in calves [52]. Flow cytometry revealed that only a scarce proportion of the CD8+/NCR1- lymphocytes expressed the γδ TCR1 suggesting that the vast majority of these cells were conventional CD8 T lymphocytes. The increase of CD8+ T cells at the onset of oocyst shedding may indicate that the adaptive immune response was initiated in the gut tissue at that time.

As the NCR1+ cell proportion within the lymphocyte population is rather low (5% on average) and the individual variability is high in very young animals, the variations due to infection were less conspicuous for NK cells than for CD8 T lymphocytes.

However, exploring the functional potential of NK cells we found that they are likely participating in the early response to this infection. The cytotoxic potential of NCR1+ lymphocytes was explored through their perforin content. Importantly, the proportion of perforin+ cells within the total NCR1+/CD8+ NK cell population, already higher than in the CD8+/NCR1- T cell population at homeostasis, increased from day 3 of infection. The higher cytotoxicity of purified jejunal and JPP NCR1+ cells from a 6 dpi-infected lamb compared to its control (Drouet unpublished data, Additional file 6) is in agreement with these data. Moreover, NCR1+ cells expressed high levels of the IL2 receptor (CD25 MFI) especially in infected lambs and their percentage was particularly high in JPPs, indicating that NCR1+ NK cells are activated in all the gut and especially in this inductive lymphoid tissue. In contrast, the proportion of perforin+ cells remained stable within the CD8+/NCR1- T cell population at 6 dpi. Analysing the cytotoxicity of CD8 T lymphocytes derived from healthy cryptosporidium seropositive and negative donors sensitized in vitro with a cryptosporidium antigen, on an intestinal cell line (CaCo2) infected with the parasite, Pantenbourg et al. [31] found that those from the seropositive donor were more cytotoxic. Comparing our in vivo results with those of Pantenbourg et al. we may hypothesize that in the present study, at 6 dpi, the CD8 T lymphocytes are likely still in the situation of a primary response in which they have a poor cytolytic potential because they are not yet fully sensitized to the parasitic antigens. Supporting this hypothesis, the CD8+ T cells were also globally less activated (low CD25 MFI) than the NCR1+ cells and surprisingly the level of expression of CD8 is not correlated with a higher activation status since CD8^hi^ T cells expressed less CD25 than the whole CD8+ population. CD8^hi^/NCR1- cells may represent cells at a different stage of differentiation.

The expression of the CD16 marker has long been associated with enhanced cytotoxic properties in human NK cells. Concerning the expression of the CD16 marker on lamb NCR1+ NK cells, we confirmed in week-old lambs the presence of CD16- and CD16+ subpopulations similar to those we have previously described in the gut of older lambs [33,49] and adults [34] and in which there was a tendency to a higher representation of the CD16+/NCR1+ NK subpopulation in older lambs [49]. The *C. parvum* infection in this study was associated with an increase in the frequency of the NCR1+/CD16+ subpopulation among total NK cells, which could indicate a higher cytotoxicity of intestinal NK cells in the infected lambs. Therefore we sought to find out if the CD16+ subpopulation displayed a higher perforin content, reflecting a higher cytotoxic potential, but the proportion of perforin+/NCR1+ lymphocytes increased in both CD16- and CD16+ subpopulations (up to 60%) with infection, especially in the jejunum. Interestingly, a cytotoxicity assay probe trial performed with CD16-/NCR1+ and CD16+/NCR1+ FACS-sorted subpopulations, failed to show any difference between the two subpopulations (Drouet unpublished data). These data suggest that the expression of CD16 on sheep NK cells might be associated with a function different from that of their human homologs, perhaps more linked with antibody dependent cell cytotoxicity than direct cytotoxicity.

Finally, considering the cytokine profile during the first days of infection, the up-regulation of IFNγ gene expression observed in inoculated lambs, is in agreement with our data obtained from rodent neonates where an early production of IFNγ is known to be a determinant for the resolution of the infection in neonatal mice [32]. Both cNK cells and T lymphocytes are known to be significant producers of IFNγ and it will be of interest to ascertain in future studies their relative contributions to its production during the course of *C. parvum* infection and resolution in sheep. The increased expression of the IL22 gene observed in all segments of the small intestine of the inoculated lambs from the very first days of infection, could come from NK22/ILC22 cells (ILC3) and/or lymphoid tissue inducers (LTi) known to produce this cytokine in mice and humans [48]. However, a recent publication [61] shows that cNK cell depletion in mouse leads to a decreased production of IL22 in the lung during *Klebsiella pneumoniae* infection, suggesting that mucosal cNK may also produce IL22. As in mouse, ovine NCR1+ cells isolated from jejunum (magnetic sorting) were able to up-regulate the expression of the IL22 gene upon in vitro stimulation by rh IL23 [50]. When more antibodies cross reactive with sheep become available, it will be of great interest to further analyse the NCR1+ together with CD8+ NCR1- lymphocytes, to study their crosstalk and characterize their subsets and respective participation in the cytokine production and resolution of cryptosporidiosis.

In the mouse model the involvement of NK cells is debated; whereas works from the team of MacDonald [19,62] support an involvement of NK1.1+ NK cells in the protective response, we recently failed [18] to demonstrate a potent role for NCR1+ NK cells in the immune response of neonatal mice to the infection with *C. parvum.* The use of different markers to analyse NK cells makes it difficult to compare results from different studies but, more importantly, the distribution of the different lymphoid cell types within the gut mucosa of neonates differs notably between ruminants and mice. The dramatic difference in their developmental status at birth is illustrated by the presence of very scarce NK cells in the gut of mice [18] and rat [44,63], while we have shown here that they are already well represented in the gut of neonatal lambs.

Altogether, the experimental data presented here demonstrate that activated NK cells that possess a high cytotoxic potential are present very early in the small intestine and likely involved in the innate response to this infection. We recently demonstrated in neonatal mice [18] that dendritic cells play a key role in the resolution of *C. parvum* infection. Dendritic cells are known to produce both IL23 and IL12 that contribute to the production of IL22 by ILC22, and IFNγ by activated cNK cells, respectively. The crosstalk between NK cells and dendritic cells warrants further study since cooperation between these two cell types, as described for other infections in human and murine species, is a key mechanism governing innate immunity against intracellular parasites. The adaptive immune response is often required for complete control of infection [18,64]. The early increase in the CD8 + T cell population that we observed in infected lambs probably coincides with the onset of the adaptive immune response and further studies are needed for better comprehension of cell interactions during this important stage of the immune response. As a specific acquired immune response requires processing and presentation of antigens to effector cells, it would be interesting to investigate the sampling of cryptosporidium antigens from the gut lumen by FAE and dendritic cells; indeed, in this study, we did not observe any cryptosporidium labelling below the epithelium, contrary to what Landsverk [29] and Åkesson et al. [65] observed with other pathogens. More specific studies on presentation of the parasite antigens to lymphocytes in the GALT would therefore be useful.

## Additional files

Additional file 1:
**Primer sequences. For PCR experiments, primer pairs were designed using Primer 3 software.** Crypto = *Cryptosporidium parvum.*
Additional file 2:
**Expression of the NCR1 gene in the gut during infection.** At slaughter, fragments of the small intestine were frozen in nitrogen and processed for quantification of the expression of the NCR1 gene by qRT-PCR. The individual data represent the ratio of the number of gene copies to the mean number of copies of two control animals slaughtered at birth. Control lambs (open symbols), inoculated lambs (filled symbols). Additional file 3:
**Absolute numbers of NCR1+ lymphocytes in the small intestine.** Cells extracted from jejunal Peyer’s patches (JPP) and jejunum from matched pairs of lambs were purified on density gradients and the numbers of MNC and lymphocyte percentages were determined by flow cytometry on morphology parameters as shown in Figure 1I. The MNC absolute number, the lymphocyte percentage and the NCR1+ cell percentage of lymphocytes were determined for each pair of lambs at 3 or 6 dpi to calculate the absolute number of NCR1+ lymphocytes (log 10 transformed) in the tissues of each animal. The matched colour symbols correspond to paired lambs; control lambs (open symbols), inoculated lambs (filled symbols). The paired *t* test performed on log 10 transformed data from JPP at day 6 pi was significant (** *p* < 0.01). Additional file 4:
**Gamma delta lymphocytes in the gut lamina propria.** Cells isolated from jejunal Peyer’s patches (JPP) and jejunum, were double labelled with anti TCR1 and CD8 mAbs. Cells with lymphocyte morphology were gated. The plots show data from a 7 day-old lamb at 6 dpi and its age-matched control. Representative plot of 3 matched pairs of 7 day-old lambs. The percentages indicated in red represent the percentage of positive cells in the dotted rectangle minus the percentage of non-specific labelling obtained with the isotype control antibody. Additional file 5:
**Expression of CD25 on small intestinal NCR1+ lymphocytes.** The expression of the activation marker CD25 was analyzed in the NCR1+ population and gated as indicated in Figure 8. The individual percentages and the mean fluorescence intensity (MFI) of CD25+ cells are shown in jejunal Peyer’s patches (JPP) and jejunum. Medians are shown with black bars. The matched color symbols correspond to paired lambs; control lambs (open symbols), inoculated lambs (filled symbols). A paired *t* test was performed on CD25-MFI data. Statistically significant differences are indicated with ** *p* < 0.01. Additional file 6:
**Cytotoxicity of small intestinal NCR1+ cells from a** ***C. parvum*** **infected lamb and its control.** At six days post-inoculation, the NCR1+ cells were isolated from Jejunal Peyer’s patches (JPP) and jejunum from an inoculated lamb and its age-matched control by magnetic sorting. The isolated cells were cultured for 4 days in the presence of recombinant ovine IL2 and recombinant human IL15. Their cytotoxicity was assessed against the ovine fibroblast line IDO5 as described by Elhmouzi et al. [51].

## Abbreviations

MLN: Mesenteric lymph node
GALT: Gut associated lymphoid tissues
IPP: Ileal Peyer’s patch
JPPs: Jejunal Peyer’s patches
FAE: Follicle associated epithelium
MNC: Mononuclear cell
NK cells: Natural killer cells
IL: Interleukin
IFNγ: Gamma interferon
CD: Cluster of differentiation
dpi: Days post-inoculation
