# Supplementary material for: The early intestinal immune response in experimental neonatal ovine cryptosporidiosis is characterized by an increased frequency of perforin expressing NCR1+ NK cells and by NCR1− CD8+ cell recruitment
Source: Vet Res. 2015 Mar 11;46:28. doi: 10.1186/s13567-014-0136-1 (PMC4355373; doi:10.1186/s13567-014-0136-1)
Supplement: Additional file 1: — Primer sequences. For PCR experiments, primer pairs were designed using Primer 3 software. Crypto = Cryptosporidium parvum. [file 13567_2014_136_MOESM1_ESM.pptx]

## Slide 1
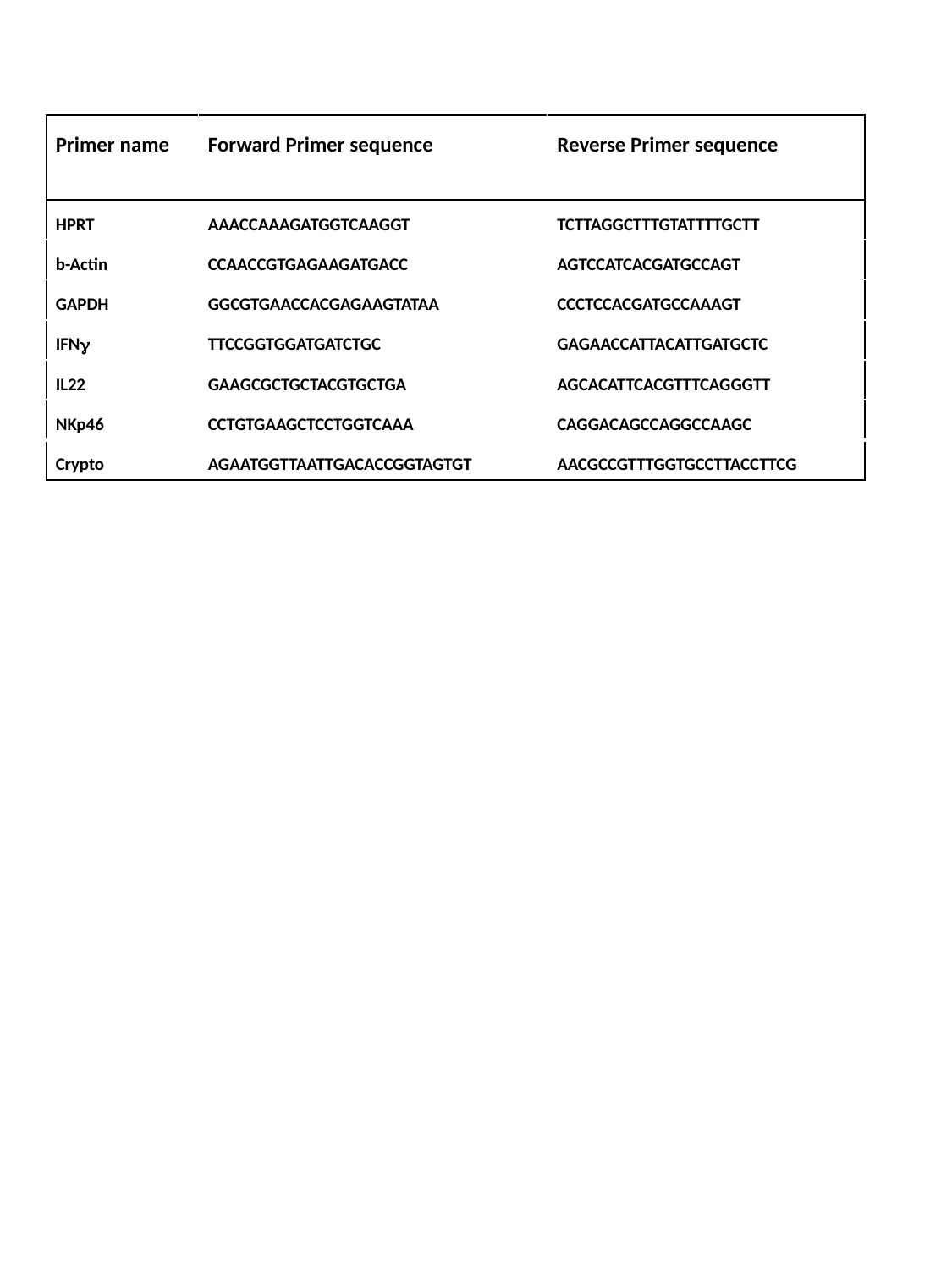

| Primer name | Forward Primer sequence | Reverse Primer sequence |
| --- | --- | --- |
| HPRT | AAACCAAAGATGGTCAAGGT | TCTTAGGCTTTGTATTTTGCTT |
| b-Actin | CCAACCGTGAGAAGATGACC | AGTCCATCACGATGCCAGT |
| GAPDH | GGCGTGAACCACGAGAAGTATAA | CCCTCCACGATGCCAAAGT |
| IFNγ | TTCCGGTGGATGATCTGC | GAGAACCATTACATTGATGCTC |
| IL22 | GAAGCGCTGCTACGTGCTGA | AGCACATTCACGTTTCAGGGTT |
| NKp46 | CCTGTGAAGCTCCTGGTCAAA | CAGGACAGCCAGGCCAAGC |
| Crypto | AGAATGGTTAATTGACACCGGTAGTGT | AACGCCGTTTGGTGCCTTACCTTCG |
