# Supplementary figures and images for: The early intestinal immune response in experimental neonatal ovine cryptosporidiosis is characterized by an increased frequency of perforin expressing NCR1+ NK cells and by NCR1− CD8+ cell recruitment
Source: Vet Res. 2015 Mar 11;46:28. doi: 10.1186/s13567-014-0136-1 (PMC4355373; doi:10.1186/s13567-014-0136-1)

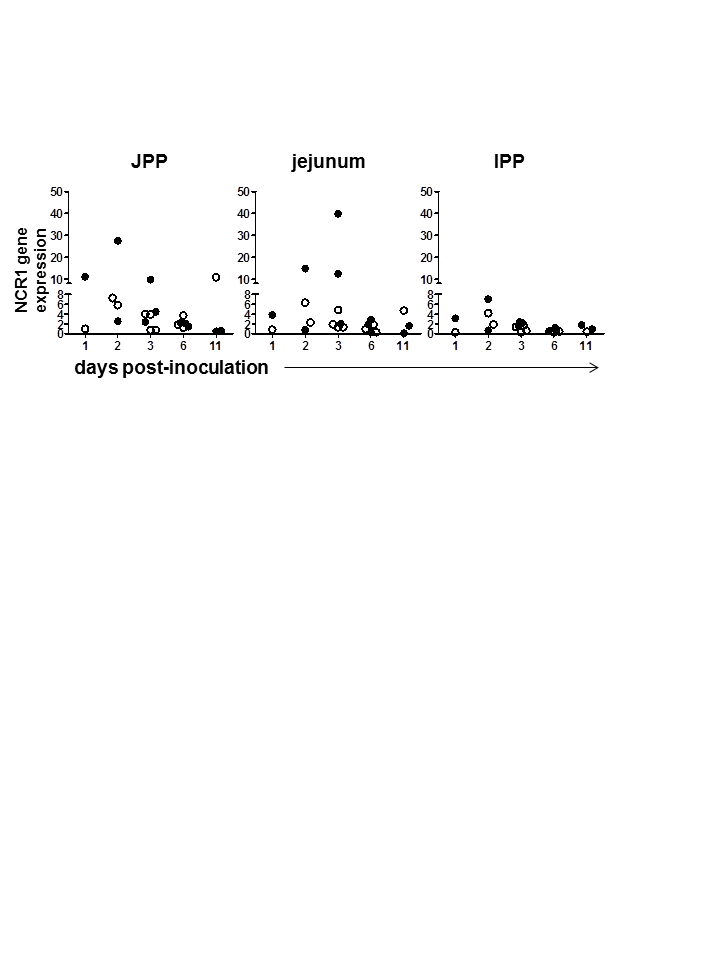

Supplement: Additional file 2: — Expression of the NCR1 gene in the gut during infection. At slaughter, fragments of the small intestine were frozen in nitrogen and processed for quantification of the expression of the NCR1 gene by qRT-PCR. The individual data represent the ratio of the number of gene copies to the mean number of copies of two control animals slaughtered at birth. Control lambs (open symbols), inoculated lambs (filled symbols). [file 13567_2014_136_MOESM2_ESM.tiff]

## Slide 1
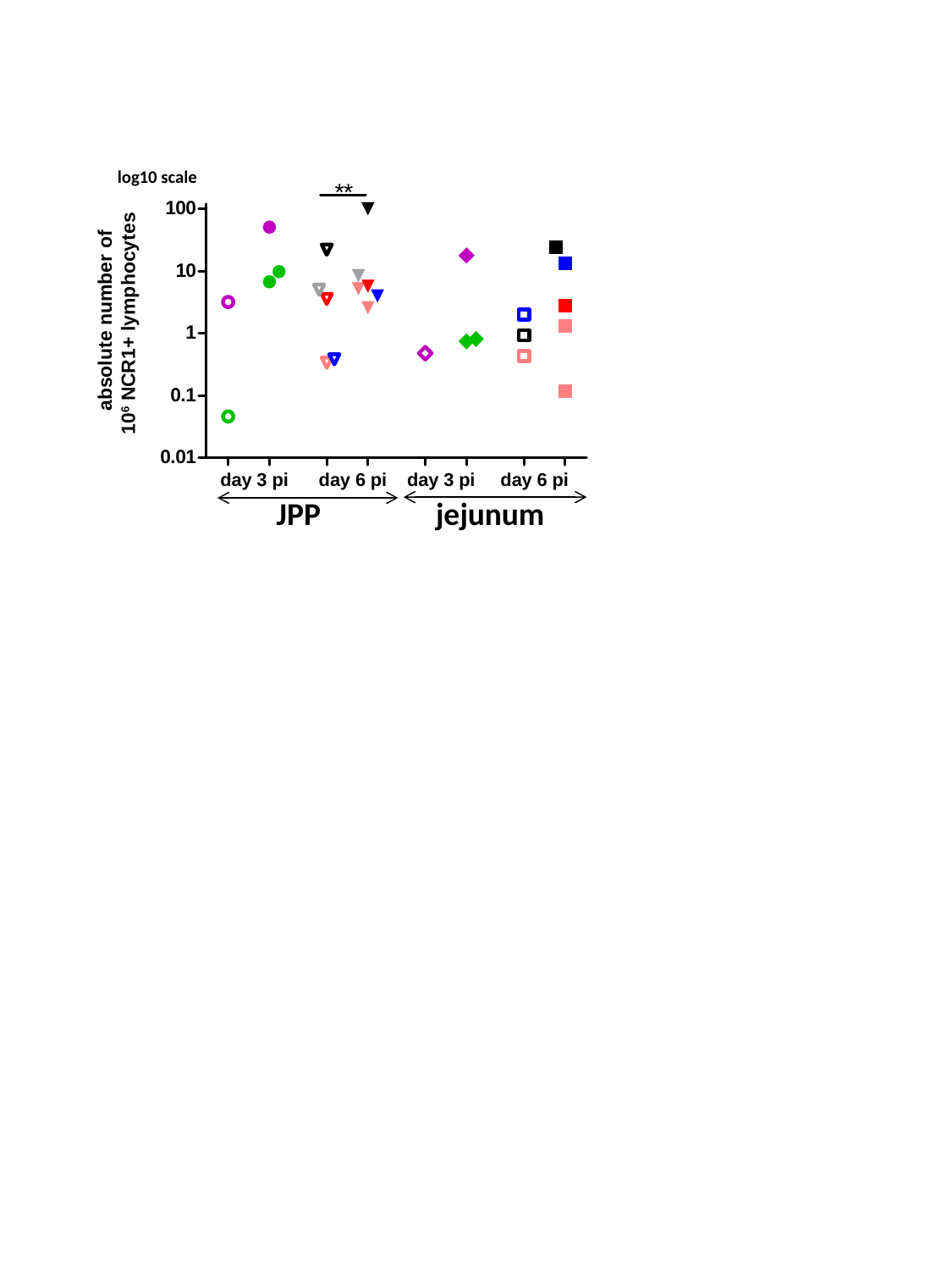

log10 scale
 absolute number of
106 NCR1+ lymphocytes
 day 3 pi day 6 pi day 3 pi day 6 pi
 JPP jejunum

Supplement: Additional file 3: — Absolute numbers of NCR1+ lymphocytes in the small intestine. Cells extracted from jejunal Peyer’s patches (JPP) and jejunum from matched pairs of lambs were purified on density gradients and the numbers of MNC and lymphocyte percentages were determined by flow cytometry on morphology parameters as shown in Figure 1I. The MNC absolute number, the lymphocyte percentage and the NCR1+ cell percentage of lymphocytes were determined for each pair of lambs at 3 or 6 dpi to calculate the absolute number of NCR1+ lymphocytes (log 10 transformed) in the tissues of each animal. The matched colour symbols correspond to paired lambs; control lambs (open symbols), inoculated lambs (filled symbols). The paired t test performed on log 10 transformed data from JPP at day 6 pi was significant (** p < 0.01). [file 13567_2014_136_MOESM3_ESM.pptx]

## Slide 1
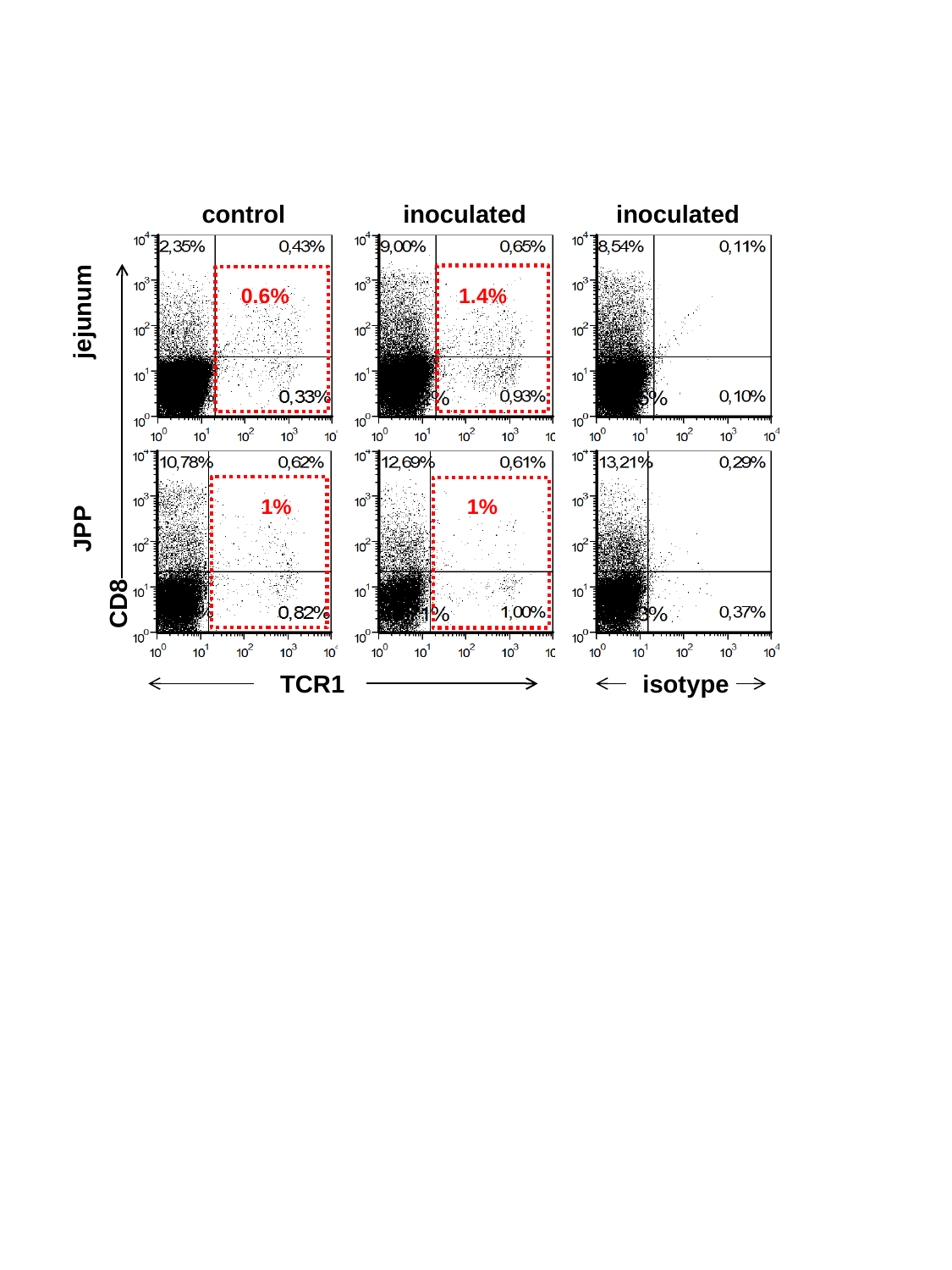

control
inoculated
inoculated
 JPP jejunum
0.6%
1.4%
1%
1%
CD8
TCR1
isotype

Supplement: Additional file 4: — Gamma delta lymphocytes in the gut lamina propria. Cells isolated from jejunal Peyer’s patches (JPP) and jejunum, were double labelled with anti TCR1 and CD8 mAbs. Cells with lymphocyte morphology were gated. The plots show data from a 7 day-old lamb at 6 dpi and its age-matched control. Representative plot of 3 matched pairs of 7 day-old lambs. The percentages indicated in red represent the percentage of positive cells in the dotted rectangle minus the percentage of non-specific labelling obtained with the isotype control antibody. [file 13567_2014_136_MOESM4_ESM.pptx]

## Slide 1
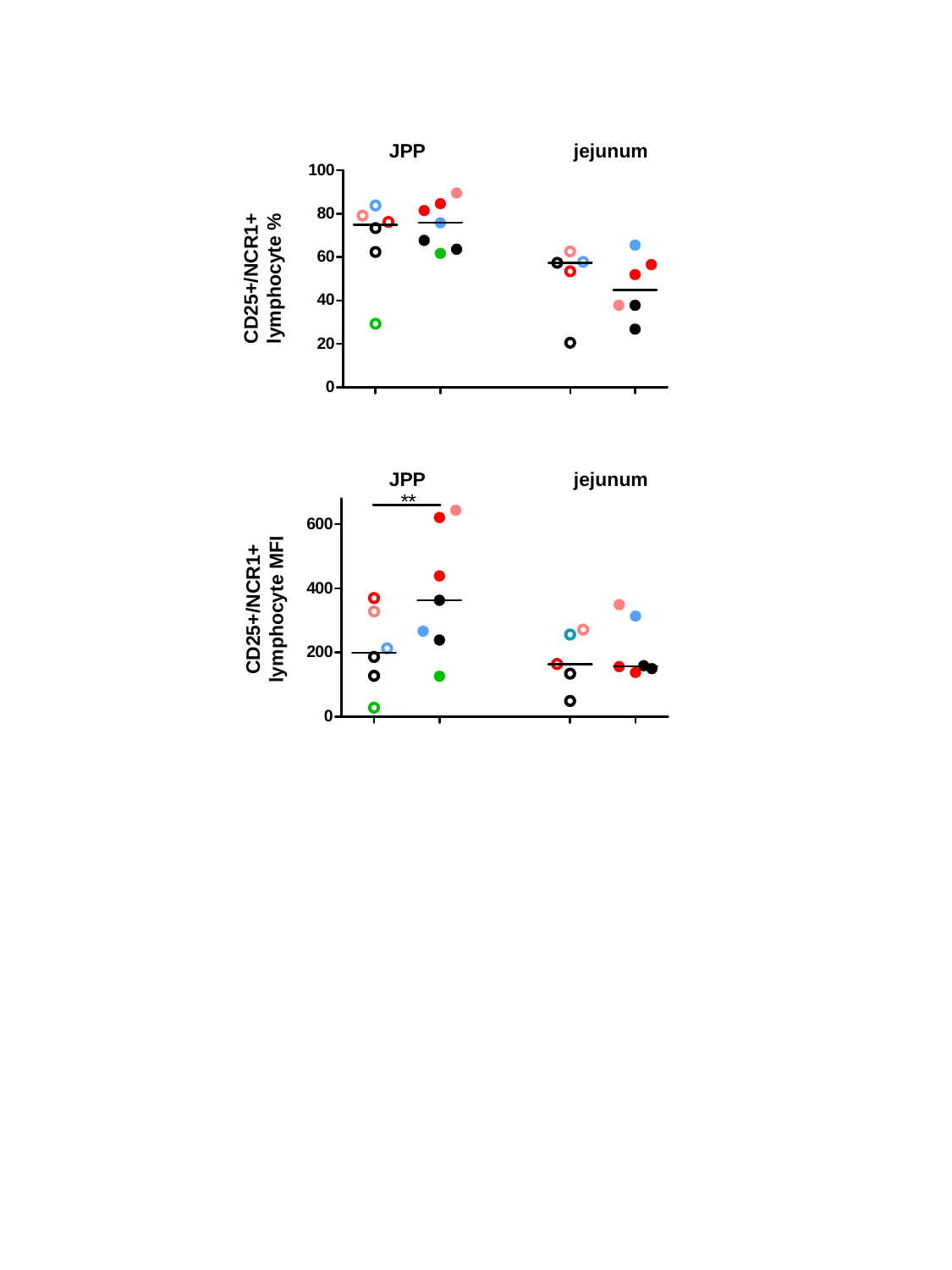

JPP jejunum
CD25+/NCR1+ lymphocyte %
 JPP jejunum
CD25+/NCR1+ lymphocyte MFI

Supplement: Additional file 5: — Expression of CD25 on small intestinal NCR1+ lymphocytes. The expression of the activation marker CD25 was analyzed in the NCR1+ population and gated as indicated in Figure 8. The individual percentages and the mean fluorescence intensity (MFI) of CD25+ cells are shown in jejunal Peyer’s patches (JPP) and jejunum. Medians are shown with black bars. The matched color symbols correspond to paired lambs; control lambs (open symbols), inoculated lambs (filled symbols). A paired t test was performed on CD25-MFI data. Statistically significant differences are indicated with ** p < 0.01. [file 13567_2014_136_MOESM5_ESM.pptx]

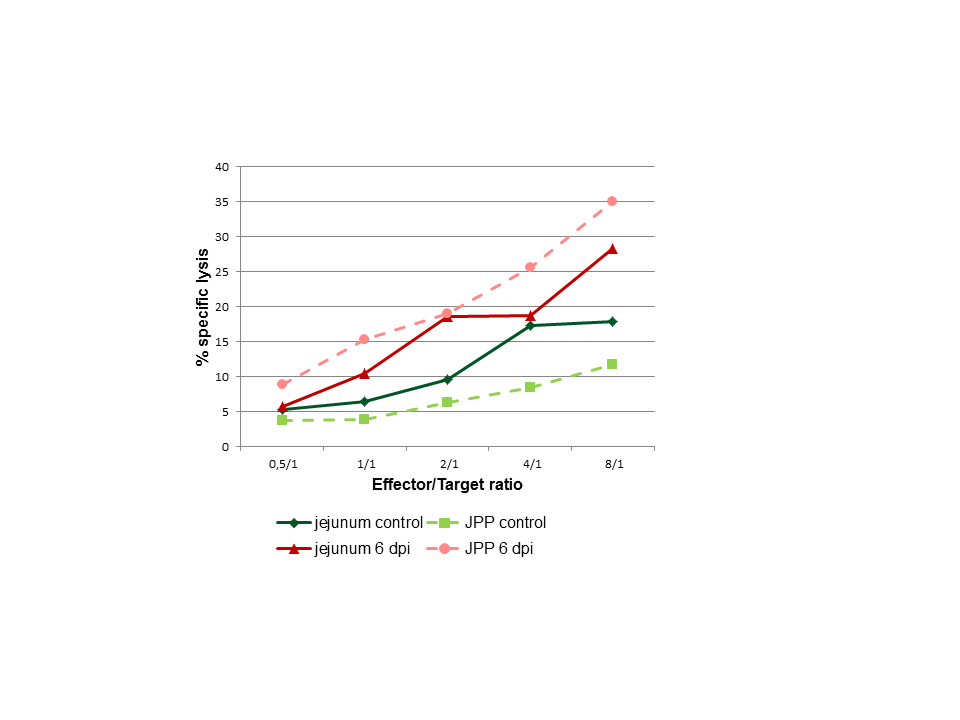

Supplement: Additional file 6: — Cytotoxicity of small intestinal NCR1+ cells from a C. parvum infected lamb and its control. At six days post-inoculation, the NCR1+ cells were isolated from Jejunal Peyer’s patches (JPP) and jejunum from an inoculated lamb and its age-matched control by magnetic sorting. The isolated cells were cultured for 4 days in the presence of recombinant ovine IL2 and recombinant human IL15. Their cytotoxicity was assessed against the ovine fibroblast line IDO5 as described by Elhmouzi et al. [51]. [file 13567_2014_136_MOESM6_ESM.tiff]
